# Impaired micro-online motor learning processes in individuals with severe anxiety

**DOI:** 10.3389/fnhum.2026.1757737

**Published:** 2026-03-23

**Authors:** Shikha Prashad, Ashley Murray, Victoria M. Rednoske

**Affiliations:** 1 Department of Kinesiology, University of Texas at Arlington, Arlington, TX, United States; 2 Department of Kinesiology and Educational Psychology, Washington State University, Pullman, WA, United States; 3 Prevention Science Graduate Program, Washington State University, Pullman, WA, United States; 4 Department of Human Development, Washington State University, Pullman, WA, United States

**Keywords:** anxiety, cognitive load, implicit motor learning, micro-offline learning, micro-online learning

## Abstract

Anxiety is a prevalent emotional state with physiological and psychological manifestations that can disrupt cognitive processes, such as working memory and attention, leading to impaired motor performance and may also influence motor learning. This study investigated the effect of anxiety on implicit motor learning, visuospatial short-term and working memory, and perceived mental and physical effort. We collected data from 79 individuals using the serial reaction time task, the Corsi block-tapping test, and the NASA-Task Load Index. Participants were grouped based on their anxiety levels measured via the Beck Anxiety Inventory. All groups demonstrated intact implicit motor learning; however, learning processes differed between groups. The severe anxiety group exhibited learning after two-minute breaks between blocks (i.e., micro-offline learning), but did not demonstrate micro-online learning (i.e., improvements in performance while executing the task). In contrast, the minimal anxiety group exhibited both learning processes. Additionally, participants with severe anxiety reported greater frustration and feeling more rushed during the task compared to those with minimal anxiety. Furthermore, the severe anxiety group also demonstrated significantly lower working memory capacity compared to their short-term memory capacity. Together, these results suggest that while individuals with higher anxiety levels demonstrated intact implicit motor learning, they required greater cognitive resources for the learning to occur.

## 1 Introduction

Motor sequence learning underpins the motor tasks we perform every day (e.g., walking up a flight of stairs or making breakfast) and those we complete for the first time (e.g., building a new desk or operating a new kitchen appliance). This process may involve declarative knowledge (i.e., explicit learning), but motor sequence learning can also occur implicitly (Destrebecqz and Cleeremans, 2001; Nissen and Bullemer, 1987; Reber, 1989). The acquisition of a novel motor skill progresses rapidly in the initial stage, with substantial improvements in performance within the first session (Censor et al., 2012; Dayan and Cohen, 2011; Doyon and Benali, 2005). This performance improvement is driven by two processes that boost performance as the task is practiced (i.e., micro-online learning) and during short breaks from the task within the session (i.e., micro-offline learning) (Bönstrup et al., 2019; Bornstein and Daw, 2012; Buch et al., 2021; Du et al., 2016; Fanuel et al., 2022; Prashad et al., 2021; Quentin et al., 2021). Together, these processes enable rapid improvements in performance during a practice session and highlight the importance of active practice and brief periods of rest for memory consolidation and motor learning.

Heightened emotional states, such as anxiety, can influence motor behavior. In an anxious state, we selectively attend to threat-related stimuli to effectively respond to a threat (Fox et al., 2005). When we are in danger, this attentional bias, physiological response [e.g., increased heart rate (Calvo and Miguel-Tobal, 1998)], and preoccupation with negative consequences (Grupe and Nitschke, 2013) can be lifesaving. However, these responses in non-threatening situations can impair cognition (Basten et al., 2011; Bishop, 2007; Eysenck et al., 2007; Fales et al., 2008; Moran, 2016; Stout et al., 2013) and impact motor behavior (Hardy et al., 2001; Hordacre et al., 2016; Lo et al., 2019; Mullen et al., 2005; Mullen and Hardy, 2000; Sporn et al., 2020). These effects are particularly concerning given the prevalence of anxiety among adults in the United States, which has increased from 15.6% in 2019 to 18.2% in 2022 (Terlizzi and Zablotsky, 2024) with the highest frequency of symptoms present in young adults between the ages of 18 and 29 years (Goodwin et al., 2020; Terlizzi and Zablotsky, 2024).

Our understanding of the impact of anxiety on motor performance is currently unclear. In a study by Hordacre et al. (2016), participants performed a precision pinch task, during which participants were asked to quickly and accurately replicate a target force. Participants in the high-anxiety group demonstrated faster reaction and movement times, as well as better accuracy, compared to the control group (Hordacre et al., 2016), suggesting that anxiety improved motor performance. In contrast, Hardy and colleagues found comparable performance when female trampolinists performed their routine under high (i.e., 2 hours prior to a national competition) and low (i.e., 2 weeks after the competition during regular practice) anxiety conditions (Hardy et al., 2001). However, when participants received task-relevant cues from a coach (i.e., instruction to guide the technique of a specific movement just before the participant performed the movement), performance of the high-anxiety group deteriorated. Similarly, when participants performed a putting task under high-anxiety conditions while simultaneously processing instructions from a coach during the movement, their performance deteriorated compared to two other conditions: (1) a task-irrelevant condition, where they generated a random letter every second under high anxiety and (2) a control condition with no additional working memory demands (Mullen et al., 2005; Mullen and Hardy, 2000). The findings from these studies support the processing efficiency theory, which suggests that performance is comprised of effectiveness (i.e., the quality of performance) and efficiency (i.e., the amount of cognitive resources required to maintain performance). Anxiety can negatively affect efficiency, but not necessarily effectiveness. Thus, individuals with higher levels of anxiety may maintain performance, but doing so requires recruiting more cognitive resources and exerting more effort than those with lower levels of anxiety (Eysenck and Calvo, 1992). This increased effort may act as a compensatory strategy to maintain performance (Ansari and Derakshan, 2011; Eysenck and Calvo, 1992). However, when cognitive demands become too high, performance may degrade. In both studies described above, task-relevant cues may have increased working memory load beyond the capacity available under high anxiety, leading to a deterioration in performance. Attentional control theory expands on this framework by proposing that anxiety affects cognition and motor processes by disrupting the balance between the goal-directed and stimulus-driven attentional systems (Eysenck et al., 2007). This disruption impairs the ability to inhibit task-irrelevant stimuli (Berggren and Derakshan, 2014; Stout et al., 2013; Wilson et al., 2016) and shift attention to the goal, thereby further taxing working memory resources. This framework helps explain why performance costs may emerge under high cognitive load, as attentional control becomes compromised despite compensatory effort.

Recent studies have also examined how anxiety influences cortical activity during motor performance and learning. Lo and colleagues measured electroencephalography (EEG) while participants performed a dart-throwing task (Lo et al., 2019). They found that in the anxiety condition, performance deteriorated, and functional connectivity in the alpha frequency band increased. The authors suggested that this change in functional connectivity during the task indicated reduced psychomotor efficiency, which may underlie the observed deficits in performance. Sporn and colleagues explored the impact of anxiety on motor learning and reported that participants in the anxiety group exhibited elevated beta power and reduced motor variability that was associated with reduced improvement in performance during the learning phase (Sporn et al., 2020). Given that beta frequencies play a critical role in motor learning and execution (Lum et al., 2023; Pollok et al., 2014), these findings indicate that anxiety may disrupt neural processes essential for motor learning.

Therefore, the aim of this study was to investigate the impact of different levels of anxiety on micro-online and micro-offline learning processes during implicit motor learning as assessed by the serial reaction time task. Anxiety was operationalized as the severity of self-reported anxiety symptoms over the past month, rather than experimentally induced anxiety. We also measured perceived task demands using the NASA-Task Load Index and visuospatial short-term and working memory using the Corsi block-tapping test. We predicted that participants experiencing higher levels of anxiety over the past month would exhibit impaired implicit motor learning with no micro-online or micro-offline learning, report greater cognitive effort to maintain performance, and exhibit reduced short-term and working memory capacity as compared to those with lower levels of anxiety over the past month.

## 2 Materials and methods

### 2.1 Participants

Seventy-nine right-handed participants (16 male, 60 female, 1 transmale, 1 non-binary, and 1 demi girl; mean age: 21.4 ± 4.3 years) provided their personal informed consent to take part in this study in accordance with the Institutional Review Board of Washington State University. Participants were required to speak English and be between 18 and 35 years of age. Participants were excluded if they had been diagnosed with or treated for a neurological or neurodevelopmental disorder. Each participant received $10 after the completion of the study.

### 2.2 Procedures

All participants completed the Beck Anxiety Inventory (BAI) (Beck et al., 1988), a self-report measure of anxiety with 21 items related to symptoms experienced in the past month. Based on BAI scores, we organized participants into four groups: minimal (*n* = 17; mean score: 4.2 ± 2.2; score range: 0–7), mild (*n* = 22; mean score: 11.4 ± 2.6; score range: 8–15), moderate (*n* = 20; mean score: 20.4 ± 3.3; score range: 16–25), and severe (*n* = 20; mean score: 37.8 ± 10.5; score range: 26–63) according to standard cutoffs (Beck et al., 1988). Participants also completed the State–Trait Anxiety Inventory (STAI) (Spielberger, 1983), a 40-item instrument in which the first 20 questions assess state (i.e., current) anxiety levels and the following 20 questions assess trait (i.e., generalized) anxiety levels. In both the STAI and BAI, higher scores are associated with higher levels of anxiety. We selected to generate the anxiety groups based on the BAI as it more effectively assesses anxiety independent of depression (Enns et al., 1998), whereas the STAI is less capable of distinguishing between anxiety and depression symptoms (Kennedy et al., 2001).

As anxiety, depression, and stress are often co-occurring (Kessler et al., 2010; Wheatley, 1997), participants also completed the Beck Depression Inventory-II (BDI) (Beck et al., 1996) and the Perceived Stress Scale (PSS) (Cohen et al., 1983) that we included as covariates in the analyses. The BDI is a 21-item self-report inventory that measures the severity of depression in adults based on their rating of symptoms in the past 2 weeks; higher scores indicate higher levels of depression. The PSS is a 14-item self-report inventory that measures how stressful situations felt to the participant in the last month. Higher scores indicate greater perceived stress. In addition, participants completed the Cognitive Failures Questionnaire (CFQ) (Broadbent et al., 1982) where they rated the frequency of occurrence of situations related to failures in perception, memory, and motor function; higher scores are associated with a greater frequency of cognitive failures. Specifically, we were interested in the distractibility and false triggering (i.e., disruptions in the processing of sequential order of cognitive and motor actions) subscores of this assessment.

We used the serial reaction time task to assess implicit motor learning. Participants were seated in front of a 27″ computer monitor and QWERTY keyboard. Stimulus presentation and response recording were controlled by Presentation software (Neurobehavioral Systems, Inc., Berkeley, CA). In this task, four white squares were presented horizontally, where the serial position corresponded to four keys on the keyboard. We instructed participants to place their left middle finger on the letter D, left index finger on F, right middle finger on K, and right index finger on J. Participants were informed that this was a reaction time task and were instructed to press the corresponding key based on which box turned red as quickly and accurately as possible. Before starting the experimental phase, a practice block of 10 stimuli was presented. In the experimental phase, participants performed eight blocks, each consisting of 120 trials. The first block was a baseline block (B0), in which the stimuli were presented in a random order. The next four blocks (B1–B4) were learning blocks consisting of a 12-item sequence that repeated 10 times in each block. Block 5 (B5) consisted of 120 trials of stimuli occurring in a random order and Block 6 (B6) consisted of the same assigned sequence as B1–B4. Since we were assessing implicit motor learning, we did not inform participants that the stimuli appeared in a repeating sequence in some blocks. Lastly, Block 7 (B7) consisted of a different sequence, constructed from the same underlying structure as the assigned sequence, to assess transfer of learning. Participants were given a mandatory two-minute unstructured break between each block. Participants’ reaction times and accuracy were recorded for each trial.

To assess whether practice with the repeating sequence resulted in explicit awareness, participants completed a post-task assessment where they answered the following question: “The stimulus movement is best described as:” with the following options: “(a) Random; (b) Some positions occurred more often than others; (c) The movement was often predictable; (d) The same sequence of movements would often appear; and (e) The same sequence of movements occurred throughout the entire experiment” (Curran, 1997). Responses were ordinally scored from 1 (random) to 5 (the same sequence of movements occurred throughout the entire experiment). Next, participants completed the NASA Task Load Index (NASA-TLX) (Hart and Staveland, 1988), a self-reported measure of perceived workload during the task in five domains: mental demand, physical demand, temporal demand, effort exerted, frustration, and perception of successful performance.

To assess visuospatial short-term and working memory capacity, participants completed a computerized Corsi block-tapping test (Corsi, 1972). In this task, nine blue squares appeared on the screen. In each trial, the squares changed to yellow one by one in a sequence. Participants were instructed to memorize the order in which the blocks changed color. All participants completed a practice block before starting the task. In the forward condition, after the presentation of the sequence, participants were required to use the computer mouse to select the boxes in the order in which they were presented to measure short-term memory. In the backward condition, participants selected the boxes in the reverse order from which they were presented to measure working memory. In both conditions, the task began with a two-item sequence and progressed to a maximum of nine items based on performance. For each condition, the greatest number of items a participant accurately recalled was their Corsi span, which represented their visuospatial short-term and working memory capacity.

### 2.3 Data analyses

For the serial reaction time task, we excluded reaction times greater than or less than 2.5 standard deviations from the mean for each participant (Ratcliff, 1993; Whelan, 2008). We also excluded reaction times for incorrect responses from the analysis and counted the number of incorrect responses for each block. We calculated mean reaction times for each block and averaged them for each group. As is standard in this task, we inferred learning through a significant reduction in reaction time from B1 to B4 (stimuli were presented in the sequential order in all four blocks), significant increase from B4 to B5 (where stimuli were presented in a random order), and significant decrease from B5 to B6 (where stimuli were presented in the same sequence as blocks B1–4). We inferred that a transfer of learning had occurred if there was a significant decrease in reaction time from B5 to B7 (Prashad et al., 2021), where stimuli were presented in a novel sequence based on the same structure as the original sequence.

To evaluate micro-online and micro-offline learning, we calculated the average reaction time for each sequence repetition (i.e., the average of every 12 reaction times as the sequence was 12 items long) in B1 through B4. Micro-online learning gain was calculated as a significant decrease in mean reaction time of the last sequence repetition in the block compared to the mean reaction time of the first sequence repetition in the same block (Fanuel et al., 2022; Prashad et al., 2021; Quentin et al., 2021). Micro-offline learning gain was operationalized as a significant decrease in mean reaction time of the first sequence repetition in a block compared to the mean reaction time of the last sequence repetition in the previous block (Buch et al., 2021; Prashad et al., 2021). The average micro-online and micro-offline learning gains across B1 through B4 were calculated for each participant and averaged across groups.

We used a 4 × 8 mixed factorial analysis of covariance (ANCOVA) with the between-subjects factor of Group (minimal, mild, moderate, and severe anxiety levels) and the within-subject factor of Block (B0–B7) on reaction time. We included BDI and PSS scores as covariates. We performed pairwise comparisons of the contrasts of interest (i.e., B1 vs. B4, B4 vs. B5, B5 vs. B6, and B5 vs. B7) that were determined *a priori*. We performed a similar ANCOVA on accuracy. To compare differences in micro-online and micro-offline learning between the groups, we used a 4 × 2 mixed factorial ANCOVA with Group (minimal, mild, moderate, and severe anxiety levels) as the between-subjects factor and Learning (micro-online and micro-offline learning) as the within-subjects factor on learning gains, controlling for BDI and PSS scores. We conducted a 4 × 2 mixed factorial ANCOVA on forward and backward Corsi span scores to assess differences in visuospatial short-term and working memory, respectively. To evaluate group differences in perceived mental and physical demand of the serial reaction time task, we conducted one-way ANCOVAs on NASA-TLX subscale ratings. We used a Bonferroni correction in all *post hoc* tests to account for multiple comparisons. We wrote custom scripts in MATLAB (MathWorks, Natick, MA) and SPSS (IBM, Armonk, NY) to analyze the data.

## 3 Results

### 3.1 Demographics

Table 1 provides an overview of the demographic and socio-cognitive assessments. There were no significant differences between the groups in age, *F*(3, 78) = 2.0, *p* = 0.12, *η*^2^ = 0.074, gender, *F*(3, 78) = 2.5, *p* = 0.063, *η*^2^ = 0.092, or educational attainment, *F*(3, 78) = 0.64, *p* = 0.59, *η*^2^ = 0.025 (see Table 1).

**Table 1:** Participant demographics (mean ± SD).

Variable	Anxiety groups				*p*-value
	Minimal	Mild	Moderate	Severe	
BAI	4.2 ± 2.2	11.4 ± 2.6	20.4 ± 3.3	37.8 ± 10.5	<0.001*
Age	23.7 ± 6.0	21.1 ± 4.2	20.6 ± 2.8	20.9 ± 3.9	0.12
Sex	11 female, 6 male	18 female, 4 male	16 female, 4 male	15 female, 2 male, 3 not listed^a^	0.063
Educational attainment (years)	14.3 ± 3.6	14.2 ± 1.9	14.1 ± 1.5	14.1 ± 2.0	0.99
BDI	3.9 ± 3.9	8.2 ± 6.2	14.6 ± 6.1	24.1 ± 10.1	<0.001*
PSS	16.7 ± 8.0	22.8 ± 6.3	31.1 ± 6.8	36.1 ± 7.2	<0.001*
STAI					
State	28.8 ± 8.1	35.2 ± 9.3	44.6 ± 7.8	54.0 ± 14.7	<0.001*
Trait	31.2 ± 8.8	41.0 ± 9.5	53.0 ± 8.9	60.8 ± 10.1	<0.001*
CFQ total score	27.9 ± 14.1	34.4 ± 11.4	47.6 ± 13.6	65.4 ± 17.2	<0.001*
Forgetfulness	11.6 ± 6.3	13.4 ± 4.2	16.8 ± 5.0	21.4 ± 5.8	<0.001*
Distractibility	10.0 ± 4.8	12.8 ± 3.6	17.6 ± 5.3	23.2 ± 5.3	<0.001*
False triggering	7.2 ± 4.6	8.6 ± 5.3	11.2 ± 4.9	17.9 ± 7.1	<0.001*

a Three participants identified as transmale, non-binary, and demi girl; BAI, Beck Anxiety Inventory; BDI, Beck Depression Inventory; PSS, Perceived Stress Scale; STAI, State–Trait Anxiety Inventory; CFQ, Cognitive Failures Questionnaire. *Significance level of *p* < 0.05.

There was a significant difference between the groups in their BDI score, *F*(3, 78) = 28.0, *p* < 0.001, *η*^2^ = 0.53, PSS score, *F*(3, 78) = 26.7, *p* < 0.001, *η*^2^ = 0.52, STAI state anxiety subscore, *F*(3, 78) = 20.6, *p* < 0.001, *η*^2^ = 0.45, and STAI trait anxiety subscore, *F*(3, 78) = 36.0, *p* < 0.001, *η*^2^ = 0.59. *Post hoc* analyses revealed a significantly higher BDI score in the severe anxiety group compared to the other groups (all *p* < 0.001, moderate, Cohen’s *d* = 1.1, mild, Cohen’s *d* = 1.9, minimal, Cohen’s *d* = 2.5). The moderate anxiety group had a significantly higher BDI score compared to the mild (*p* = 0.044, Cohen’s *d* = 0.98) and minimal (*p* < 0.001, Cohen’s *d* = 1.9) anxiety groups. There was no significant difference in BDI score between the mild and minimal anxiety groups (*p* = 0.40, Cohen’s *d* = 0.81). The severe anxiety group also reported a significantly higher PSS score compared to the mild (*p* < 0.001, Cohen’s *d* = 1.8) and minimal (*p* < 0.001, Cohen’s *d* = 2.4) anxiety groups, but not the moderate anxiety group (*p* = 0.63, Cohen’s *d* = 0.51). The moderate anxiety group had a significantly higher PSS score compared to the mild (*p* = 0.001, Cohen’s *d* = 1.4) and minimal (*p* < 0.001, Cohen’s *d* = 2.0) anxiety groups. There was no significant difference in PSS score between the minimal and mild anxiety groups (*p* = 0.059, Cohen’s *d* = 0.87). The severe anxiety group also exhibited significantly higher STAI state and trait anxiety subscores compared to the minimal (state, *p* < 0.001, Cohen’s *d* = 2.1; trait, *p* < 0.001, Cohen’s *d* = 3.1), mild (state, *p* < 0.001, Cohen’s *d* = 1.5, trait, *p* < 0.001, Cohen’s *d* = 2.0), and moderate (only state anxiety was significantly different, state, *p* = 0.041, Cohen’s *d* = 0.79; trait, *p* = 0.061, Cohen’s *d* = 0.82) anxiety groups. The moderate anxiety group had significantly higher STAI state and trait anxiety subscores compared to the minimal (state, *p* < 0.001, Cohen’s *d* = 1.1; trait, *p* < 0.001, Cohen’s *d* = 2.5) and mild (state, *p* = 0.020, Cohen’s *d* = 2.0; trait, *p* < 0.001, Cohen’s *d* = 1.3) anxiety groups. The mild anxiety group exhibited higher STAI trait, but not state, anxiety subscores than the minimal anxiety group (state, *p* = 0.58, Cohen’s *d* = 0.64; trait, *p* = 0.015, Cohen’s *d* = 1.0).

There was also a significant difference between the groups in the distractibility, *F*(3, 73) = 4.7, *p* = 0.005, *η*^2^ = 0.16 and false triggering, *F*(3, 73) = 4.8, *p* = 0.004, *η*^2^ = 0.16, but not forgetfulness, *F*(3, 73) = 2.1, *p* = 0.10, *η*^2^ = 0.081 subscores of the CFQ when controlled for BDI and PSS. For distractibility, the severe anxiety group reported a significantly higher subscore compared to the minimal (*p* = 0.019, Cohen’s *d* = 2.5), mild (*p* = 0.004, Cohen’s *d* = 2.5), and moderate (*p* = 0.026, Cohen’s *d* = 2.3) anxiety groups. The severe anxiety group reported a significantly higher false trigger subscore compared to the mild (*p* = 0.021, Cohen’s *d* = 1.5) and moderate (*p* = 0.003, Cohen’s *d* = 1.1) anxiety groups, but not the minimal anxiety group (*p* = 0.087, Cohen’s *d* = 1.8).

### 3.2 Implicit motor sequence learning

#### 3.2.1 Accuracy

We found a significant main effect of Block, *F*(7, 511) = 2.1, *p* = 0.039, partial *η*^2^ = 0.028 on accuracy of key presses, but no main effect of Group, *F*(3, 73) = 1.1, *p* = 0.36, partial *η*^2^ = 0.043 and no significant interaction, *F*(21, 511) = 0.77, *p* = 0.75, partial *η*^2^ = 0.031. *Post hoc* analyses revealed significantly fewer errors in B1 (*p* = 0.008, Cohen’s *d* = 0.42), B4 (*p* = 0.045, Cohen’s *d* = 0.36), B6 (*p* < 0.001, Cohen’s *d* = 0.61), and B7 (*p* = 0.001, Cohen’s *d* = 0.47) compared to B0.

#### 3.2.2 Mean reaction times

**Figure 1:**
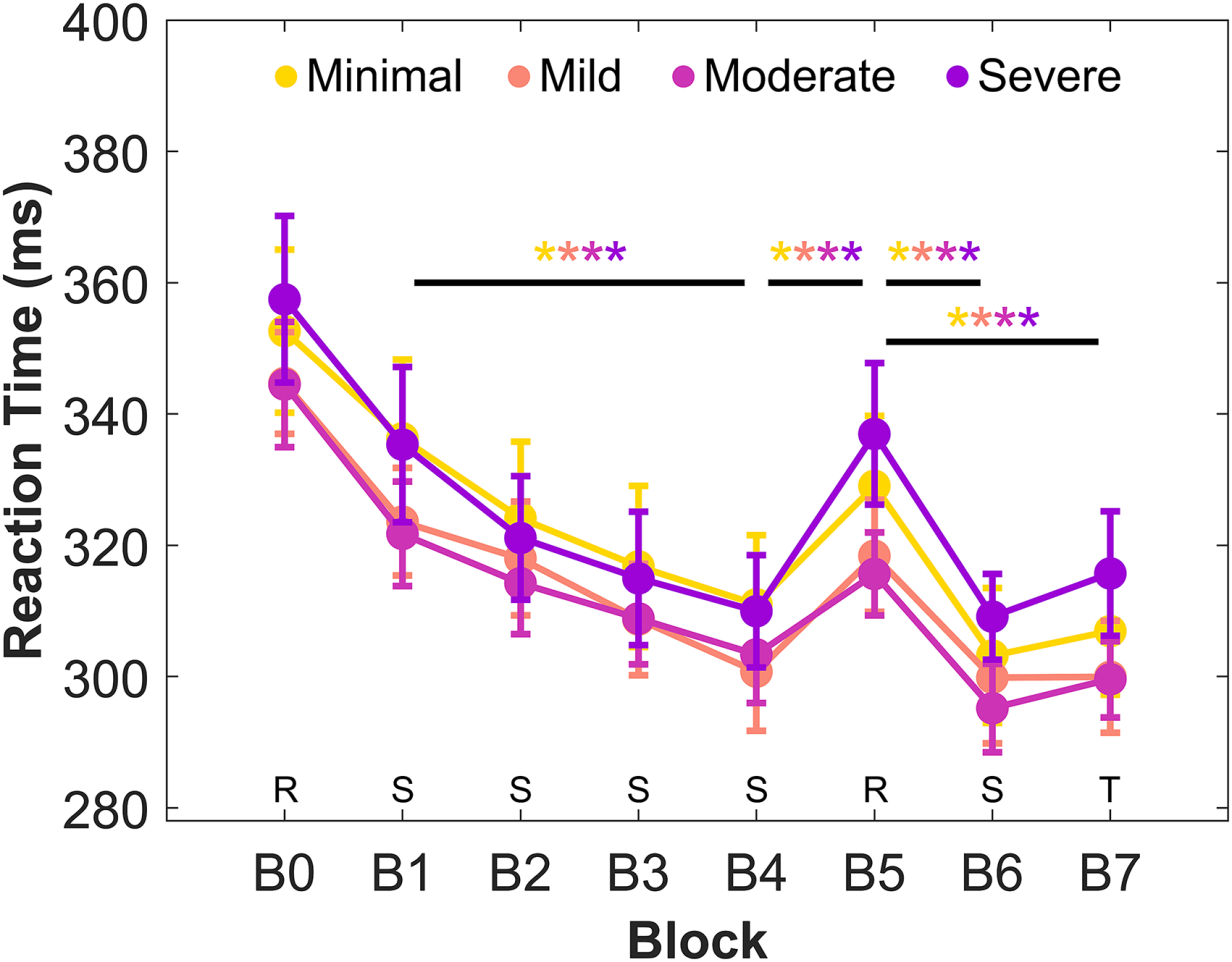
Mean reaction times of each block and group. Stimuli were presented in a random order in B0 and B5 (i.e., R), in a sequential order of the assigned sequence in B1–4 and B6 (i.e., S), and in a sequential order of a novel sequence in B7 (i.e., T). Error bars indicate standard error. * Significance level of *p* < 0.05.

There was a main effect of Block, *F*(7, 511) = 3.5, *p* = 0.001, partial *η*^2^ = 0.046, but no main effect of Group, *F*(3, 73) = 0.31, *p* = 0.82, partial *η*^2^ = 0.013 and no significant interaction, *F*(21, 511) = 0.72, *p* = 0.82, partial *η*^2^ = 0.029 on reaction times. Bonferroni corrected *post hoc* analyses on contrasts of interest that were determined *a priori* revealed a significant decrease in reaction time from B1 to B4 in all groups (minimal, *p* < 0.001, Cohen’s *d* = 1.4; mild, *p* < 0.001, Cohen’s *d* = 1.0; moderate, *p* = 0.004, Cohen’s *d* = 1.1; and severe, *p* < 0.001, Cohen’s *d* = 1.1). All groups also exhibited a significant increase in reaction time from B4 to B5 (minimal, *p* < 0.001, Cohen’s *d* = 1.5; mild, *p* = 0.001, Cohen’s *d* = 0.93; moderate, *p* = 0.015, Cohen’s *d* = 0.79; and severe, *p* < 0.001, Cohen’s *d* = 1.2), a significant decrease in reaction time from B5 to B6 (minimal, *p* < 0.001, Cohen’s *d* = 1.6; mild, *p* = 0.002, Cohen’s *d* = 0.96; moderate, *p* = 0.002, Cohen’s *d* = 1.2; and severe, *p* < 0.001, Cohen’s *d* = 1.0), and a significant decrease in reaction time from B5 to B7 (minimal, *p* < 0.001, Cohen’s *d* = 1.5; mild, *p* < 0.001, Cohen’s *d* = 1.2; moderate, *p* = 0.003, Cohen’s *d* = 0.95; and severe, *p* < 0.001, Cohen’s *d* = 1.1). These comparisons are shown in Figure 1.

#### 3.2.3 Micro-online and micro-offline learning

**Figure 2:**
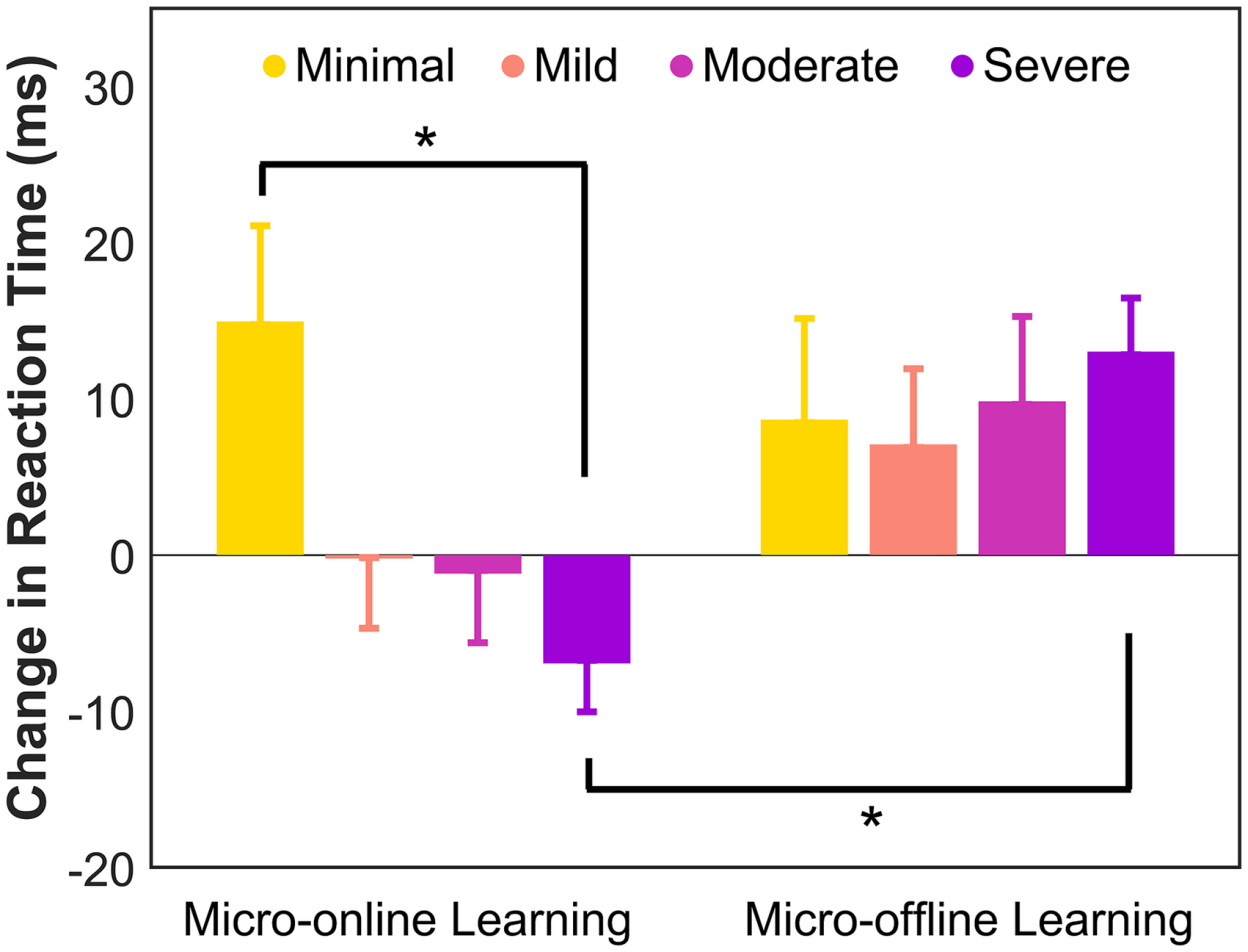
Micro-online and micro-offline learning in each group. A positive difference between reaction times reflects learning gains that indicate micro-online or micro-offline learning. Error bars indicate standard error. * Significance level of *p* < 0.05.

There was a significant main effect of Group, *F*(3, 73) = 2.7, *p* = 0.050, partial *η*^2^ = 0.099 and a significant interaction between Group and Learning, *F*(3, 73) = 1.1, *p* = 0.043, partial *η*^2^ = 0.12, but no main effect of Learning, *F*(1, 73) = 0.14, *p* = 0.71, partial *η*^2^ = 0.038. *Post hoc* analyses revealed significantly greater overall learning (collapsed across micro-online and micro-offline learning) in the minimal anxiety group compared to the mild (*p* = 0.039, Cohen’s *d* = 4.3) and severe (*p* = 0.033, Cohen’s *d* = 4.0) anxiety groups, but not the moderate anxiety group (*p* = 0.33, Cohen’s *d* = 3.8). The minimal anxiety group exhibited significantly higher micro-online gains compared to the severe anxiety group (*p* = 0.019, Cohen’s *d* = 0.93; see Figure 2), which exhibited a loss in performance. Additionally, in the severe anxiety group, there was a significantly greater gain in micro-offline learning than in micro-online learning (*p* = 0.034, Cohen’s *d* = 0.77). There were no differences between micro-online and micro-offline gains in the other groups.

#### 3.2.4 Posttest

In the posttest, there were no differences in explicit awareness of the sequence between the groups, *F*(3, 72) = 0.77, *p* = 0.51, *η*^2^ = 0.031.

#### 3.2.5 Perceived task workload

**Figure 3:**
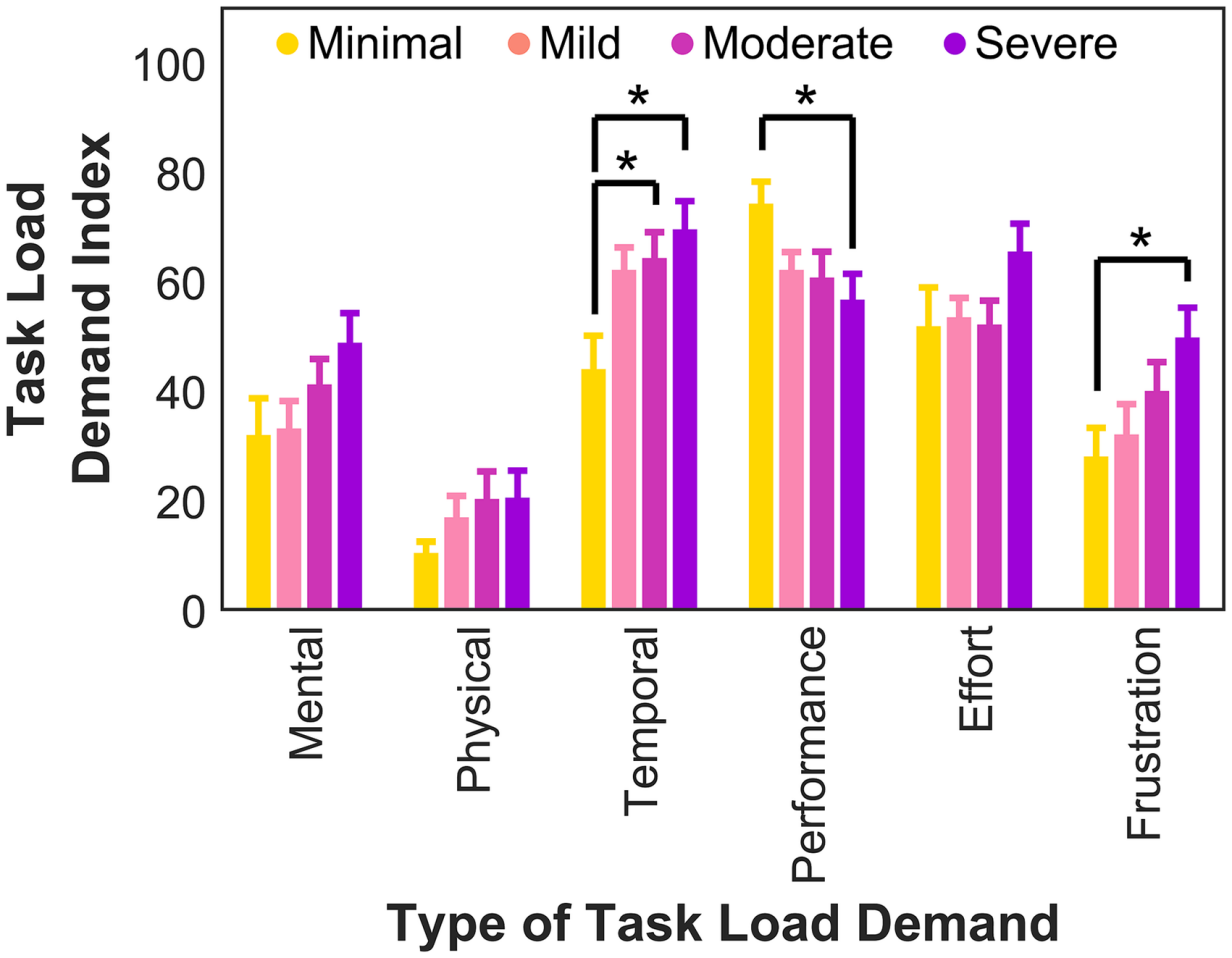
Task load demand for the serial reaction time task for each group. The performance task load was reverse coded to match the other types of task load. Error bars indicate standard error. * Significance level of *p* < 0.05.

During the serial reaction time task, there was a significant difference in perceived temporal demand, *F*(3, 73) = 4.5, *p* = 0.006, *η*^2^ = 0.15, perceived success in the performance of the task, *F*(3, 73) = 2.9, *p* = 0.041, *η*^2^ = 0.10, and frustration, *F*(3, 73) = 3.1, *p* = 0.034, *η*^2^ = 0.11. *Post hoc* tests corrected for multiple comparisons demonstrated that the minimal anxiety group reported feeling less rushed compared to the moderate (*p* = 0.041, Cohen’s *d* = 0.87) and severe (*p* = 0.005, Cohen’s *d* = 1.1) anxiety groups (see Figure 3). Compared to the minimal anxiety group, the severe anxiety group reported feeling that they were less successful in accomplishing the task (*p* = 0.036, Cohen’s *d* = 0.93) and more frustrated (*p* = 0.039, Cohen’s *d* = 0.95). There were no other significant differences between the groups. There were also no significant differences between the groups in perceived mental demand, *F*(3, 73) = 2.1, *p* = 0.11, *η*^2^ = 0.077, physical demand, *F*(3, 73) = 1.1, *p* = 0.36, *η*^2^ = 0.042, or effort expended, *F*(3, 73) = 1.7, *p* = 0.17, *η*^2^ = 0.065.

### 3.3 Visuospatial short-term and working memory

There was no significant main effect of Corsi span, *F*(1, 73) = 1.2, *p* = 0.29, partial *η*^2^ = 0.016, or Group, *F*(3, 73) = 0.90, *p* = 0.45, partial *η*^2^ = 0.036, but there was a significant interaction, *F*(3, 73) = 3.8, *p* = 0.013, partial *η*^2^ = 0.14. *Post hoc* analyses corrected for multiple comparisons revealed that working memory capacity was significantly higher in the moderate anxiety group compared to the mild anxiety group (*p* = 0.038, Cohen’s *d* = 0.72). In addition, short-term memory capacity was significantly higher compared to working memory capacity in the mild (*p* < 0.001, Cohen’s *d* = 1.3) and severe (*p* = 0.003, Cohen’s *d* = 0.99) anxiety groups. There were no other significant differences between groups.

## 4 Discussion

The aim of this study was to explore the effect of different levels of anxiety over the past month on the implicit learning of a motor sequence. We found that while anxiety did not impair overall implicit motor learning, there were differences in the underlying learning processes, particularly between individuals with severe anxiety and those with minimal anxiety. While the minimal anxiety group exhibited both micro-online and micro-offline gains in performance, the severe anxiety group only exhibited micro-offline learning. Participants in the severe anxiety group also reported feeling more rushed and frustrated while completing the serial reaction time task and their scores on failures related to cognition indicated increased distractibility and disruptions in processing sequential cognitive and motor actions. Finally, we found no differences in visuospatial short-term memory among the groups, but the moderate anxiety group had significantly higher working memory capacity compared to the mild anxiety group. In addition, those in the mild and severe anxiety groups demonstrated significantly lower working memory capacity compared to short-term memory capacity. Thus, despite preserved overall implicit motor learning, our findings suggest that elevated levels of anxiety may alter the way individuals navigate the learning process.

Across all levels of anxiety, we found improvements in motor performance in the serial reaction time task and intact learning of the deterministic motor sequence. These improvements in performance were observed through faster reaction times at the end of the four blocks of practice. Learning was inferred by a significant increase in reaction time when exposed to randomly presented stimuli after consecutive practice with the sequence. Although these findings contrast with studies suggesting that anxiety disrupts cognitive resources critical for motor learning (Eysenck et al., 2007; Eysenck and Calvo, 1992; Moran, 2016; Shackman et al., 2006) and impacts motor performance (Hardy et al., 2001; Lo et al., 2019; Mullen and Hardy, 2000) and learning (Sporn et al., 2020), they align with attentional control theory which proposes that performance can be maintained when individuals with anxiety use compensatory strategies.

While overall implicit motor learning was preserved, differences emerged in the underlying motor learning processes between the severe and minimal anxiety groups, suggesting that individuals with severe anxiety may require increased recruitment of cognitive resources to maintain overall performance. We found an absence of micro-online learning (i.e., improvement in performance during the task) in the severe anxiety group. While participants in the minimal anxiety group demonstrated micro-online gains while performing the task, those with severe anxiety exhibited slower reaction times as they performed the task. However, participants in the severe anxiety group maintained overall performance by achieving micro-offline gains after the two-minute breaks between blocks. Micro-online learning consists of updating each item of the sequence with each trial (Bornstein and Daw, 2012, 2013; Verstynen et al., 2012), which can be taxing on working memory. Thus, maintaining performance throughout a block may require greater cognitive resources, potentially leading to declines in performance within the practice blocks, and the absence of micro-online learning. Interestingly, this pattern (i.e., an absence of micro-online learning coupled with intact micro-offline learning) resembles learning dynamics that have been observed for more complex probabilistic motor sequences (Prashad et al., 2021) that impose higher cognitive demands. Further, the severe anxiety group self-reported significantly higher scores in distractibility and disruptions in the processing of sequential cognitive and motor actions. This association between higher scores in these factors of cognitive failures is consistent with prior literature (Dzubur et al., 2020; Goodhew and Edwards, 2024; Mahoney et al., 1998) and may also have contributed to the absence of micro-online learning. We found additional support for a more strenuous learning process through perceived task workload. The severe anxiety group reported feeling more frustrated and rushed while completing the serial reaction time task compared to the minimal anxiety group. Feeling rushed can indicate that the task was placing increased demands on cognitive resources and required more effortful performance (Castellotti et al., 2022; D’Agostino et al., 2023; Polti et al., 2018; Yang et al., 2025). Taken together, these findings suggest that while severe anxiety did not eliminate implicit motor learning, it altered the learning trajectory and may have reduced processing efficiency, consistent with attentional control theory’s proposal that anxiety impairs efficiency even when performance is maintained through compensatory strategies (Ansari and Derakshan, 2011; Eysenck et al., 2007).

Based on prior research, we expected that higher levels of anxiety would be associated with reduced working memory capacity (Moran, 2016; Shackman et al., 2006; Vytal et al., 2013). However, this hypothesis was not supported in our sample, as we only found a significant difference in working memory capacity between the moderate and mild anxiety groups. One possible explanation for this discrepancy may be that the Corsi block-tapping task primarily measures visuospatial short-term and working memory, which may be less susceptible to the effects of anxiety than tasks that require verbal working memory (Derakshan and Eysenck, 1998; Eysenck et al., 2005; MacLeod and Donnellan, 1993; Moran, 2016). Although some studies report anxiety-related impairments in spatial working memory (Lavric et al., 2003; Shackman et al., 2006; Vytal et al., 2013), evidence also suggests that these effects are context-dependent and more pronounced under conditions of high cognitive load (Eysenck et al., 2007). For example, individuals with high levels of anxiety exhibited performance declines on verbal reasoning (Derakshan and Eysenck, 1998; MacLeod and Donnellan, 1993) and spatial and verbal *n*-back (Vytal et al., 2013) tasks when working memory demands were high. Our findings may also indicate that the Corsi block-tapping task was not sufficiently complex to tax cognitive resources significantly, and therefore anxiety did not exert a strong enough influence to produce group differences. Consistent with this observation, studies have found that without additional load on working memory, individuals with high anxiety levels may perform similarly to those with low anxiety levels (Lukasik et al., 2019; MacLeod and Donnellan, 1993). Interestingly, we found that in the mild and severe anxiety groups, working memory capacity was significantly lower than their short-term memory capacity, suggesting a disproportionate impact on working memory. This reduced working memory capacity may underlie the absence of micro-online learning in the severe anxiety group, as participants may have been unable to perform trial-by-trial updates to improve performance while executing the task.

These findings are best interpreted in the context of the limitations of this work. First, we found significant differences between the groups in their depression and stress scores. As depression and stress often co-occur with anxiety (Kessler et al., 2010; Wheatley, 1997), particularly within a young adult sample (Kessler et al., 2010), disentangling their unique effects on implicit motor learning remains challenging. Although we statistically controlled for depression and stress in our analyses, these factors may still exert neurological influences that may interact with anxiety and affect the results. Furthermore, as the anxiety groups exhibited significant differences in both state and trait anxiety symptoms, it remains unclear which dimension primarily accounted for the results. Future investigation might also assess how anxiety regulation strategies influence micro-online and micro-offline learning processes on individuals with elevated anxiety to ascertain whether such approaches benefit those whose symptoms impair performance. Prior studies have found that interventions such as mindfulness (Call et al., 2014; Nesrine et al., 2025), exercise (Anderson and Shivakumar, 2013; Carter et al., 2021), and yoga (Li and Goldsmith, 2012; Subramanya and Telles, 2009) can reduce anxiety symptoms and enhance cognitive functioning. These interventions may similarly support micro-online learning in individuals with higher anxiety levels. Second, a common limitation of using deterministic sequences to assess implicit motor learning is the potential for participants to become explicitly aware of the repeating sequence (Meissner et al., 2016; Press et al., 2005). However, our post-test assessment revealed little awareness of a repeating pattern and no differences between groups, indicating that the learning remained implicit. Third, the greater micro-online gains observed in the minimal anxiety group may partly reflect increased attentional engagement during the task rather than differences in implicit learning *per se*. According to attentional control theory, anxiety can redirect attention to task-irrelevant stimuli (Berggren and Derakshan, 2014; Eysenck et al., 2007; Stout et al., 2013; Wilson et al., 2016) potentially disrupting goal-directed processing and contributing to reduced micro-online gains in individuals with severe anxiety. Future research may examine the role of attentional control in implicit motor learning among individuals with varying levels of anxiety. Finally, while we did not find that elevated levels of anxiety had greater deficits in visuospatial short-term and working memory capacity, individual differences in these domains may still influence implicit motor learning. Future research may address these limitations by using tasks that vary in complexity and cognitive load. For example, probabilistic sequences and dual-task paradigms can both impose higher cognitive demands that may further elucidate the impact of anxiety on implicit motor learning. Incorporating neuroimaging methods, such as electroencephalography (EEG) or functional magnetic resonance imaging (fMRI), during the serial reaction time task may further elucidate whether anxiety disrupts neural dynamics underlying implicit motor learning, including potential differences in neural patterns during micro-online and micro-offline learning.

In sum, we found that while anxiety did not affect overall implicit learning of a deterministic motor sequence, it altered *how* learning occurred. Individuals with minimal anxiety demonstrated intact micro-online learning, as their performance improved as they practiced the deterministic sequence, and intact micro-offline learning, as their performance also improved after the two-minute breaks between blocks. In contrast, those with severe anxiety relied on micro-offline learning. This difference in learning processes suggests that the severe anxiety group may not have had sufficient cognitive resources to support micro-online learning. Further, the severe anxiety group subjectively rated the task as feeling more frustrating and rushed than the minimal anxiety group. Together, these findings indicate that elevated levels of anxiety may produce a cognitively challenging learning environment, where maintaining performance may come at the cost of increased cognitive load. Pragmatically, these findings may be applied to various learning situations. Providing regular, short breaks throughout the learning process may allow individuals with elevated levels of anxiety to clear their cognitive workspace and allow for learning to occur.

## Data Availability

The raw data supporting the conclusions of this article will be made available by the authors, without undue reservation.
